# Clinical problems of computer-guided implant surgery

**DOI:** 10.1186/s40902-016-0063-3

**Published:** 2016-03-24

**Authors:** Seong-Yong Moon, Kyoung-Rok Lee, Su-Gwan Kim, Mee-Kyoung Son

**Affiliations:** 1 Department of Oral and Maxillofacial Surgery, School of Dentistry, Chosun University, 375 Seosuk dong, Dong-gu, Gwangju, 501-759 South Korea; 2 Department of Prosthodontics, Graduate School of Dentistry, Chosun University, 375 Seosuk dong, Dong-gu, Gwangju, 501-759 South Korea; 3 Department of Prosthodontics, School of Dentistry, Chosun University, 375 Seosuk dong, Dong-gu, Gwangju, 501-759 South Korea

**Keywords:** Implant, CT-guided surgery, Computer-assisted surgery, Angular errors, Distance errors

## Abstract

**Background:**

The utilization of a cone-beam computed tomography (CT)-assisted surgical template allows for predictable results because implant placement plans can be performed in the actual surgery. In order to assess the accuracy of the CT-guided surgery, angular errors and shoulder/apex distance errors were evaluated by data fusion from before and after the placement.

**Methods:**

Computer-guided implant surgery was performed in five patients with 19 implants. In order to analyze differences of the implant fixture body between preoperative planned implant and postoperative placed implant, angular error and distance errors were evaluated.

**Results:**

The mean angular errors between the preoperative planned and postoperative placed implant was 3.84° ± 1.49°; the mean distance errors between the planned and placed implants were 0.45 ± 0.48 mm horizontally and 0.63 ± 0.51 mm vertically at the implant neck and 0.70 ± 0.63 mm horizontally and 0.64 ± 0.57 mm vertically at the implant apex for all 19 implants.

**Conclusions:**

It is important to be able to utilize these methods in actual clinical settings by improving the various problems, including the considerations of patient mouth opening limitations, surgical guide preparation, and fixation.

## Background

For successful implants, the anatomical limitations of patients need to be understood, and the necessity of additional surgeries, including bone grafts, should be confirmed prior to the surgery. Thus, preoperative diagnoses and the establishment of treatment plans with expectations of the esthetic and functional aspects of the final implant prostheses are essential [1, 2]. In the past, implant surgical templates have been prepared in models, which are separate from the computed tomography (CT) data and which have been used only as a position indicator for reference of the placement position during the surgery [1–4], thereby providing surgical information only on placement position. This technique has the limitation of not providing information on placement depth. However, with the development of novel software, surgical guides can be prepared by the direct utilization of the CT data. Therefore, it is possible to prepare 3D surgical templates that can place an implant at the planned position and depth prior to surgery. With the increased use of this technique due to the various advantages of computer-guided implant surgery, a variety of studies have been conducted on the accuracy after the placements. This study aimed to present the clinical issues in actual guided surgeries through the examination of the cases of patients that were treated with computer-guided implant surgery and confirm the accuracy of guided surgery by analyzing whether the positions before and after the placement matched.

## Methods

**Fig. 1:**
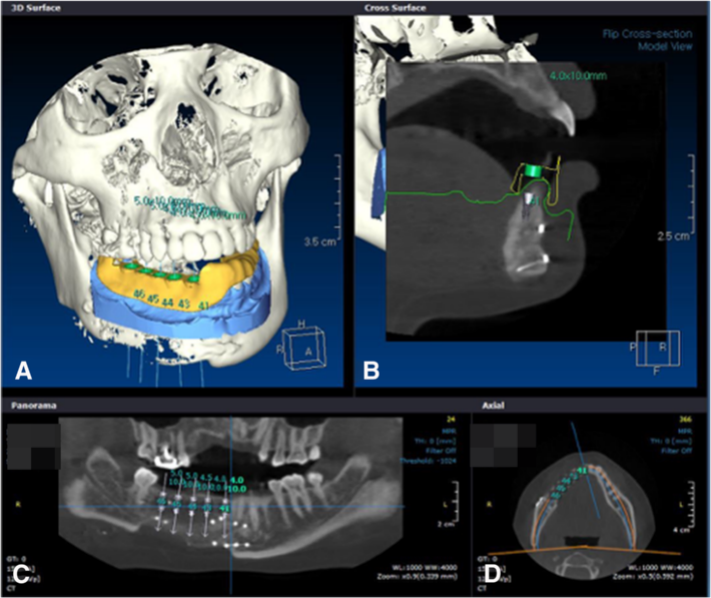
Five implants were placed in the simulation software at the time of preoperative planning. **a** Surgical template was designed in the software. **b** Surgical template and sleeve were seen in cross section. **c** Five implants’ locations were seen in panoramic CT view. **d** Implants were seen in axial view

**Fig. 2:**
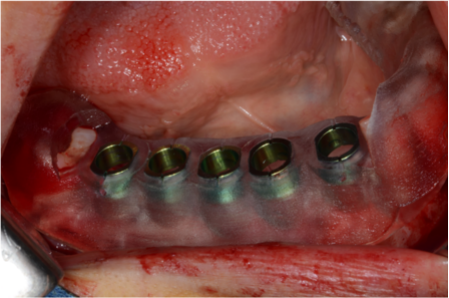
Fabricated surgical template

**Fig. 3:**
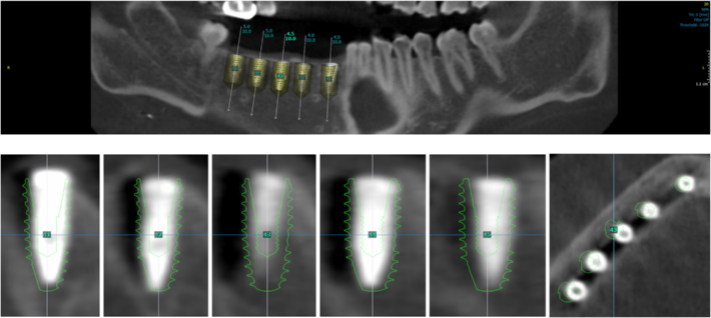
Fused image for the comparison of the preoperative implant position and the postoperative implant position

**Fig. 4:**
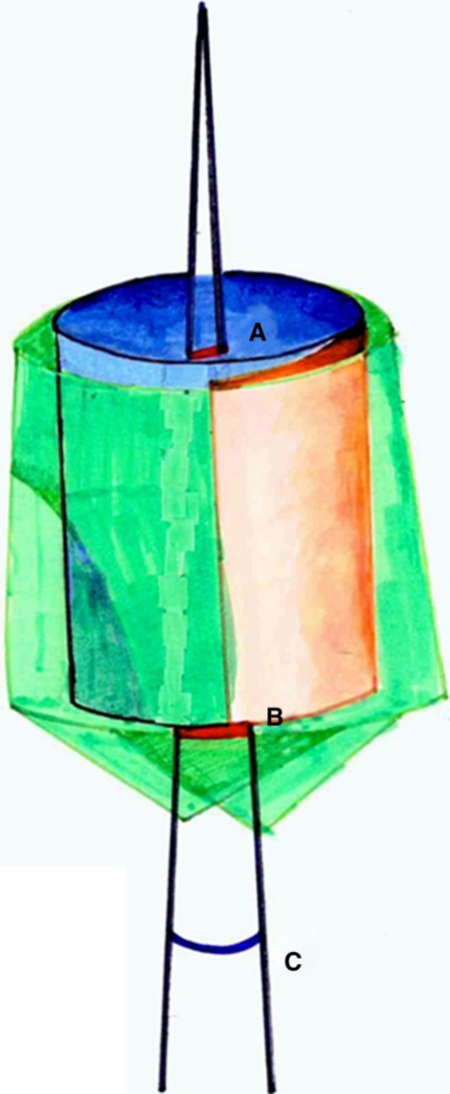
The angular and distance (shoulder/apical) error analysis of the preoperative and postoperative implant fixture body. **a** The distance error (mm) of the fixture center at the implant shoulder area. **b** The distance error (mm) of the fixture center at the implant apex area. **c** The angular error (°) of the implant fixtures [5]

The study protocol was approved by the institutional ethics committee of the Chosun University Dental Hospital. Written informed consent was obtained from all of the patients. Computer-guided implant surgery was performed in five patients with 19 implants. Surgical guides were prepared with different methods for the partial edentulous and full edentulous patients. The partial edentulous patients had residual teeth other than the defect, and, therefore, data that were obtained from a stone model that was made by taking an impression in the patient was fused with the 3D scanned data and the CT scanned data in accordance with the teeth. The positions of the implant placement were planned, and the data were sent to a preparation center to fabricate a surgical template (Fig. 1). Because fusion of the teeth is impossible for full edentulous patients, a radiographic stent needed to be prepared. Holes were formed on the prepared stent at regular intervals. After radiation-impermeable materials, such as gutta percha, were filled, two different CT scans were performed. Firstly, a radiographic stent was installed in the patients for acquiring the patient’s own images, and then the CT scan data of the radiograghic stent only was acquired. Then, those two images were fused. For the standard of the fusion, the radiation-impermeable holes that were formed on the radiographic stent were used in the data fusion instead of teeth. For both partial edentulous and full edentulous patients, data fusion, implant placement position and angle determination, and data transmission were done with In2guide^®^ software (Cybermed, Seoul, Korea) (Fig. 1). The implant placement plans were then established, and the data were sent to a preparation center to prepare a surgical template. The surgical template was prepared with stereolithography (SLA)-assisted rapid prototyping (RP). In SLA (stereolithography), a liquid photopolymerized resin performs a laminate charge of 1 mm with a laser. The SLA machine recognizes the diameter and angle of the simulated implants and selectively polymerizes the resins around it, and this is followed by the formation of a cylindrical guide in accordance with the corresponding implant. Then, a metal sleeve is attached to the cylindrical guide to guide the implant drill. Consequently, the angulation and mesiodistal and buccolingual positioning of the implant that was planned with the 3D computer simulation software was transferred to a SLA surgical guide in order to form a surgical template. In the present cases, a Dentis haptite^®^ fixture (Dentis, Deagu, Korea) was placed with a Dentis surgical guide drill set (Fig. 2). After the implant placement, the data that were obtained from the CT scan were fused with the planned data before the implant placement from the computer software (Fig. 3). In order to analyze differences of the implant fixture body between preoperative planned implant and postoperative placed implant, angular error and distance errors were evaluated (Fig. 4) [5].

## Results

All 19 implants were installed in five patients. Eleven implants were placed using tooth-supported surgical guides for partial edentulous patients, and eight implants were installed using mucosa-supported surgical guides in fully edentulous patient. The mean angular errors between the preoperative planned implant and postoperative placed implant was 3.84° ± 1.49°; the mean distance errors between the planned and placed implants were 0.45 ± 0.48 mm horizontally and 0.63 ± 0.51 mm vertically at the implant neck and 0.70 ± 0.63 mm horizontally and 0.64 ± 0.57 mm vertically at the implant apex for all 19 implants (Table 1).

**Table 1:** Angular and distance errors of the preoperatively planned implant position and the postoperative implant position

Patient no. (edentulous state)	Position	Angular error (°)	Shoulder error (mm)			Apical error (mm)		
			Horizontal error		Vertical error	Horizontal error		Vertical error
			dx	dy	dz	dx	dy	dz
1 (Partial)	#36	6,74	0.1	0.91	0.33	0.4	2.05	0.36
	#37	4.43	0.48	0.72	0.3	0.1	1.39	0.38
2 (Partial)	#36	4.1	0.29	0.02	0.33	0.4	0.16	0.26
	#37	4.32	0.46	0.09	0.62	1.04	0.58	0.66
3 (Partial)	#25	2.44	0.41	0.17	1.07	0.5	0.58	1.15
	#26	7.88	0.95	1.02	1.86	1.44	2.3	2.02
4 (Partial)	#41	0.71	0.71	0.42	0.49	0.71	0.42	0.49
	#42	3.05	1.18	0.2	0.09	1.18	0.2	0.09
	#43	3.77	1.71	0.62	0.22	1.72	0.01	0.11
	#44	3.08	1.46	0.03	0.04	1.47	0.5	0.11
	#45	4.82	1.96	0.14	0.63	1.97	0.97	0.73
5 (Full)	#26	3.56	0.22	0.54	1.78	0.2	1.14	1.93
	#24	3.01	0.42	0.14	1.04	0.43	0.37	1.16
	#23	4.95	0.11	0.02	0.83	0.09	0.88	0.66
	#22	3.97	0.08	0.14	0.64	0.05	0.55	0.58
	#12	5.03	0.01	0.19	0.07	0	0.67	0.05
	#13	1.97	0.11	0.41	0.72	0.1	0.06	0.69
	#14	3.86	0.06	0.46	0.47	0.06	1.15	0.42
	#16	4.12	0.02	0.24	0.43	0.02	0.94	0.39
Mean ± standard deviation		3.84 ± 1.49	0.57 ± 0.61	0.34 ± 0.30	0.63 ± 0.51	0.63 ± 0.64	0.79 ± 0.62	0.64 ± 0.57
Horizontal and vertical error (mean ± standard deviation)			0.45 ± 0.48		0.63 ± 0.51	0.70 ± 0.63		0.64 ± 0.57

Horizontal error: dx and dy; vertical error: dz; distance error: dx, dy, and dz

## Discussion

Computer-guided implant surgery allows for accurate and safer implant placement and has the advantage of surgical time reduction. However, it requires CT and additional software and the additional costs of the surgical drill kit and guide preparation for the guided surgery. With the increased application of computer-guided implant surgery for accurate and predictable implant surgeries, various studies have been conducted on its accuracy. Di Giacomo et al. [6] prepared six surgical templates with a SLA method in four patients and compared the accuracy after implant placement. They showed an angular error of 7.25° ± 2.67° and a distance error of 1.45 ± 1.42 mm at the shoulder and 2.99 ± 1.77 mm at the apex. Schneider et al. [7] reported an angular error of 5°–6° and a shoulder/apex distance error of 1.07 mm/1.63 mm. In 2009, Ozan et al. implemented a guide surgery with CAD software and RP and compared the preoperative and postoperative errors. They reported that all of the implants exhibited a mean angular error of 4.1° ± 2.3° and a mean distance error of 1.11 ± 0.7 mm at the implant shoulder and 1.41 ± 0.9 mm at the implant apex. That study also compared accuracy according to the types of surgical guide supports by the range of teeth loss. Particularly for an angular error, the utilization of surgical guides as a tooth support was reported to result in a smaller angular error than that of a bone support and a mucosa support. Thus, most studies have reported that a smaller error range was observed when the guided surgery placed an implant by free hand or a surgical template was prepared with a conventional method [8–10]. The results in the cases in the present study supported the results of the above studies, with mean angular errors of 3.86° (0.71°–7.88°) in partial edentulous cases and 3.80° (1.97°–5.03°) in full edentulous cases. In implant placement, a vertical error occurs when the placement is deeper or shallower than that in the plan, and a horizontal error occurs when there is lateral displacement compared to the plan. In guided surgery, a large vertical error is more common than a large horizontal error. This is because a superior border of alveolar bone is difficult to differentiate in CT data during the implant plan. Therefore, after the actual placement, there are often cases that require slightly deeper placement after guide removal. In general, distance errors are larger in the apex than in the shoulder. This may be because a horizontal error that is due to angle displacement at an occlusal surface increases closer to the apex. A previous study of the accuracy of guided surgery with In2Guide^®^ software, which was the software employed in the present cases [5], installed 30 implants in 15 partial edentulous patients and compared the installations before and after the maxilla and mandible implants. The maxilla and mandible angular errors were 1.89° ± 1.14° and 3.93° ± 3.41°, respectively, and the occlusal distance errors were 0.63 ± 0.37 mm and 1.46 ± 0.71 mm. The apical distance errors were 0.79 ± 0.37 mm and 1.46 ± 0.68 mm. In the study, a smaller error in the maxilla compared to the mandible was explained by the observation that the maxillary surgical template was broadly supported by the teeth and palate mucous membranes. Thus, surgical template support and stability was suggested to have substantial influence on the accuracy of implant placement. In the present cases, the implants adjacent to the teeth support were more accurate than the ones without support at the most distal portion for distal extension of the posterior teeth. For distal extension, most stents obtain support from the proximal teeth, and the vertical movement of a stent without distal support can exist during placement. The factors involved in the angle and distance errors in computer-guided implant surgery include errors in data fusion due to limitations in CT resolution and metal scattering, lack of conformity of the guide due to teeth crowding or rotation, difficulties in selecting anchor pin positions for stent fixation in edentulous patients, limitations in patient mouth opening, drop out of metal sleeves, and stent fractures. Recently, a high-resolution dental CT has been reported. However, the accuracy can deteriorate during data fusion due to metal scattering issues in the presence of patient prostheses and resolution limitations of the tissues in distal extension partial edentulous and full edentulous patients, which can result in errors. When surgery is performed by installing a guide with teeth support, the superior suitability of stents can result in very accurate outcomes. However, insertion problems because of residual teeth and tissue undercuts in the installation of the removable surgical guides can cause lack of conformity of the guide and postoperative errors. Stents in edentulous patients are mostly maintained with tissue support. Thus, there can be movement. Therefore, the positions for the anchor pins to fix the movement before surgery are selected and used as a surgical fixture body. However, when an implant is closely placed, the selection of anchor pin is not feasible. In a surgical guide, a metal sleeve is mounted in the order of each drill. The metal sleeve should be a certain length in order to guide the drills and implants to their designated positions. Therefore, because a surgical template should be a certain thickness for metal sleeve filling, implant installation with a surgical template can be restricted in distal teeth and in patients with mouth opening limitations. In 2007, Hans-Joachim et al. [3] reported that the utilization of surgical template is accurate but limited due to insufficient intermaxillary distance in some cases. In clinical case 1 in this study, installation with a guide was impossible due to the lack of intermaxillary distance in the distal extension edentulous of #37. In addition, a metal sleeve allows for slight horizontal movement in order to allow drills and implants to pass so that slight placement angle and distance errors can occur, even when placement is performed with an accurate guide. In case 3, the metal sleeve dropped out during the placement and was reattached. Once attachment issues occur in the preparation of a surgical guide and metal sleeve, problems can occur in the guided surgery. Thus, attention needs to be given to the preparation, and gentle handling is requested during the surgery. When considering accurate implant placement and the patient costs and operators’ efforts in guide preparation, questions of whether patients have proper bone quality for CT-guided surgery, whether the surgery time is appropriate, and what kind of implants is planned to be used need to be confirmed prior to the surgery [3, 11]. In cases in which an initial fixation of implants is difficult due to weak bone quality during surgery, the surgical problems in the guided surgery are difficult to solve. In addition, surgical guide-induced placements can only selectively use fixture bodies from some implant companies. It is therefore necessary to confirm the possibility of guided surgery before surgery when the patients want certain implants or when a placement needs to be done with an implant with special surfaces because of patient conditions, including bone quality. Although the errors in computer-guided implant surgery are in the clinically acceptable range, they can cause complications when an implant is planned close to anatomical structures, such as the maxillary sinus and nerves. Thus, the patients’ anatomical structures should be accurately assessed before performing the surgery, with full dependence on the surgical template [5]. Moreover, for more accurate guided surgery, the abovementioned limitations of the guided surgery need to be adequately recognized and then avoided. For more accurate computer-guided implant surgery, various studies, including the development of standardized methods to reduce errors in the fusion of scanned CT, model scan data, and doubled CT data and improvements in accurate surgical template preparation methods for minimum errors, are necessary.

## Conclusions

19 implants were installed in five patients with CT-guided surgical templates, the clinical problems during surgery were analyzed, and the differences between preoperative and postoperative implant positions were evaluated. Our results were considered favorable compared to the free hand method, but various limitations were still observed. It is important to be able to utilize these methods in actual clinical settings by improving the various problems, including the considerations of patient mouth opening range, surgical guide shape, length of metal sleeve and surgical drill, template supporting problem, and scanning method.

### Consent for publication

Written informed consent was obtained from the patient for publication and any accompanying images. A copy of the written consent is available for review by the Editor-in-Chief of this journal.
